# A Shocking Outcome: Cardioversion-Induced Flash Pulmonary Edema

**DOI:** 10.7759/cureus.78249

**Published:** 2025-01-30

**Authors:** Oscar Diaz, Kevin Sande, Guillermo Loyola, Glenda Muguruza

**Affiliations:** 1 Dr. Kiran C. Patel College of Osteopathic Medicine, Nova Southeastern University, Fort Lauderdale, USA; 2 Internal Medicine, Palmetto General Hospital, Miami, USA; 3 Internal Medicine, University of Florida College of Medicine – Jacksonville, Jacksonville, USA; 4 Internal Medicine, Baptist Health South Florida, Miami, USA

**Keywords:** atrial fibrillation, cardiac electrophysiology, cardioversion, flash pulmonary edema, mechanical ventilation

## Abstract

Atrial fibrillation (AF) is the most common major cardiac rhythm disorder in adults and causes considerable morbidity and mortality, particularly in elderly patients. We present the case of a 75-year-old female admitted for acute heart failure with a history of AF and recurrent heart failure exacerbations. Despite aggressive medical management with amiodarone and diuretics, the patient continued to deteriorate. The cardiology team recommended electrical cardioversion to re-establish a normal sinus rhythm. By doing so, the patient developed flash pulmonary edema after the procedure and required immediate intubation with mechanical ventilation. There was a need for tracheostomy to facilitate prolonged ventilatory support after some time. Over time, they were weaned off the ventilator and discharged home after full recovery. This case illustrates the challenges of managing AF in an elderly patient and the possible risks of flash pulmonary edema due to cardioversion.

## Introduction

Atrial fibrillation (AF) is the most common of the serious cardiac rhythm disturbances in the adult population. It is a major public health concern due to its association with significant morbidity and mortality. The attributable risk for stroke associated with AF increases steeply from 1.5% at age 50-59 years to 23.5% at age 80-89 years [1]. This sharp increase underscores the critical need for effective management strategies, particularly in the aging population, to mitigate the substantial risk of stroke and other thromboembolic events. Under current guidelines, cardioversion is the treatment of choice commonly used to restore AF to sinus rhythm in order to facilitate the management of heart failure. If the patient is hemodynamically unstable, or if sinus rhythm has not returned after treatment with anti-arrhythmic drugs, then electric direct-current cardioversion should be performed under sedation with a short-acting anesthetic (class I recommendation) [2].

Here, we highlight the unusual instance of flash pulmonary edema following electrical cardioversion for AF treatment. An elderly female who presented with an acute exacerbation of heart failure was found to be in AF once she arrived at the emergency department. The patient did not have a prior pacemaker or a history of sick sinus syndrome. Initial imaging suggested fluid overload, which was aggressively treated without improvement. The electrophysiology team made the decision to perform electrical cardioversion, which precipitated an episode of flash pulmonary edema, requiring emergent intubation, and eventual tracheostomy.

## Case presentation

The patient is a 75-year-old woman, who had a medical history of AF and had been hospitalized multiple times for acute heart failure exacerbations. She was transported to the emergency department, where she complained of worsening shortness of breath, fatigue, and edema on her legs. These presenting symptoms were indicative of an acute exacerbation of heart failure with suspected reduced ejection fraction. Her medical records indicated a long-standing case of AF, for which she was prescribed amiodarone to regulate her heart rhythm. Despite being under current treatment, she had a prior history of multiple decompensations that resulted in hospitalizations.

**Figure 1:**
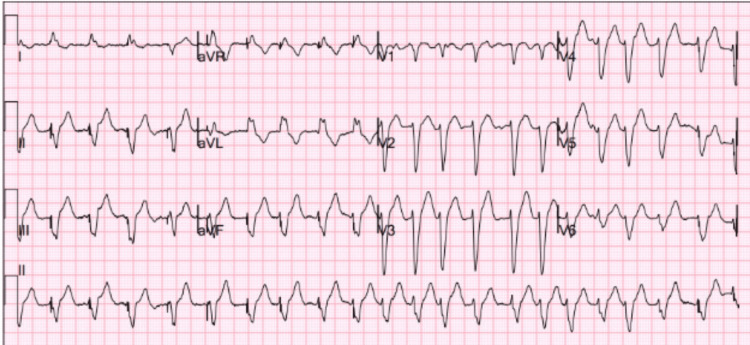
EKG showing atrial fibrillation and rapid ventricular response

**Figure 2:**
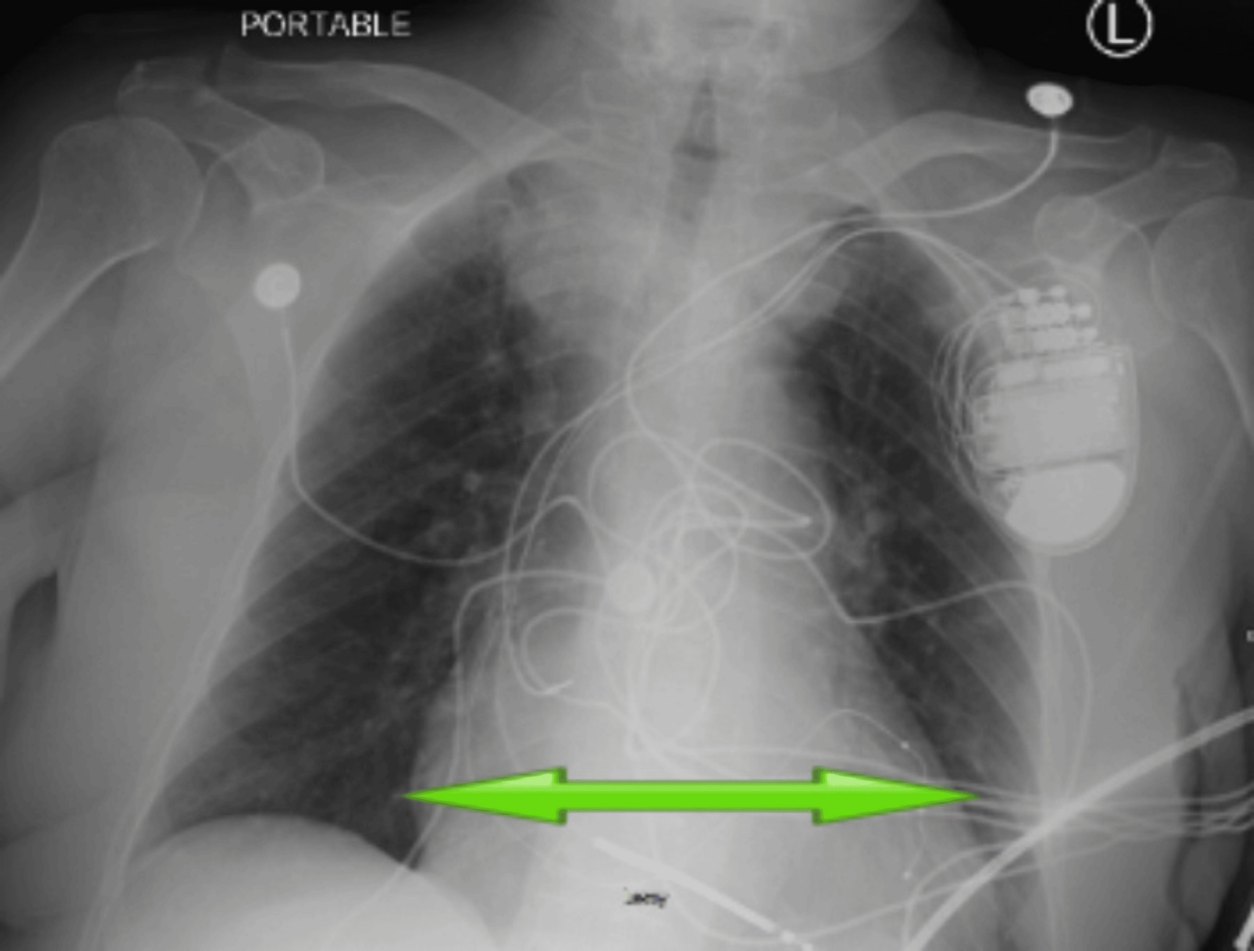
Chest X-ray on admission showing cardiomegaly

Upon admission, an electrocardiogram, shown in Figure 1, confirmed the presence of AF accompanied by a rapid ventricular response, with a heart rate of 112 beats per minute. The chest radiography findings in Figure 2 indicated considerable signs of pulmonary congestion and an enlarged heart, which aligned with her current condition of worsening heart failure. The patient's condition was addressed by administering an intravenous amiodarone drip to regulate her heart rate, in addition to utilizing non-invasive positive pressure ventilation to alleviate respiratory distress. An aggressive diuresis was started using intravenous loop diuretics in order to address the fluid overload and enhance her hemodynamic condition.

The electrophysiology team was consulted at an early stage due to her past AF and the observed connection between her arrhythmia and recurring episodes of heart failure exacerbations. Following a thorough assessment, the team suggested synchronized electrical cardioversion as a potential course of action once the patient's condition becomes stable. However, after undergoing the procedure, which was performed using 150 joules of energy, the patient did not revert back to sinus rhythm and continued displaying AF with the new development of flash pulmonary edema. This condition was described for the first time by Pickering et al. in 1988 as a clinical picture of recurrent edema of the lungs [3]. The rapid and urgent presentation required the need for intubation and mechanical ventilation to effectively address her respiratory failure.

In spite of extensive supportive measures such as high-dose diuretics and providing ventilatory support, the patient did not pass multiple attempts at spontaneous breathing in the following days. This suggested a poor outlook for successfully discontinuing mechanical ventilation. As a result of her ongoing reliance on mechanical respiratory support, a tracheostomy was carried out to aid in the long-term management of ventilation. After a week with mechanical ventilation in place, the patient was able to be successfully weaned off and was able to achieve a total recovery and be discharged home.

## Discussion

This case underscores the complexities and challenges of managing AF, particularly in elderly patients with concurrent heart failure. AF is not only the most common serious cardiac rhythm disturbance in the adult population, affecting approximately 10% of individuals by the age of 80, but it also significantly complicates the clinical management of heart failure due to its impact on hemodynamics [4]. The patient's case highlights the risks associated with electrical cardioversion, a commonly used intervention to restore sinus rhythm in patients with AF. Although cardioversion is generally effective, this case demonstrates the potential for severe complications, such as flash pulmonary edema, which in this instance led to an urgent need for intubation and mechanical ventilation.

The pathophysiology underlying flash pulmonary edema, particularly in the setting of AF and electrical cardioversion, remains not fully understood. However, it has been suggested that transient severe functional mitral regurgitation might play a role in precipitating flash pulmonary edema, although the exact mechanisms are still unclear [5]. This association between flash pulmonary edema and mitral valve dysfunction emphasizes the need for careful consideration of the risks and benefits when electing to perform cardioversion, especially in the vulnerable aging population. In this case, the development of flash pulmonary edema post-cardioversion required aggressive management and led to prolonged mechanical ventilation, ultimately necessitating a tracheostomy for long-term respiratory support.

Advanced age is a significant risk factor not only for AF but also for concomitant coronary artery disease (CAD). Studies indicate that nearly 70% of all patients with AF have underlying CAD, a condition that exacerbates the hemodynamic burden of AF and significantly worsens clinical outcomes, including mortality. Elderly patients aged >65 years with AF, with a history of one or more cardiovascular comorbidities, or evidence of atherosclerosis in other vascular beds should warrant a closer look and a dedicated effort to look for associated CAD [6]. The interplay between CAD and AF is complex, with shared risk factors such as age, hypertension, and diabetes contributing to their coexistence. Moreover, the presence of CAD often increases the severity and frequency of AF episodes, creating a vicious cycle of worsening cardiovascular health. Recent evidence suggests that the management of underlying CAD, including lifestyle interventions, optimized medical therapy, and revascularization when indicated, can reduce the burden of AF and improve overall outcomes.

Our patient's advanced age, a known risk factor for both AF and adverse outcomes, likely contributed to the complexity of her condition. Aging is associated with structural and electrical changes in the myocardium, which can lead to the development and persistence of AF, as well as complications such as heart failure and flash pulmonary edema [7]. This case illustrates the importance of a comprehensive, multidisciplinary approach to the management of AF in elderly patients, taking into account the potential for age-related changes to exacerbate the disease process.

Individualized patient care, particularly when dealing with high-risk interventions like electrical cardioversion in elderly patients with complex comorbidities, is paramount. It also highlights the importance of early recognition and prompt management of complications such as flash pulmonary edema, which remains largely a clinical diagnosis based on a careful history and physical examination [8]. The successful outcome, with the patient eventually weaning off mechanical ventilation and recovering fully, reflects the critical role of timely and appropriate interventions, as well as the resilience of the patient despite the significant challenges posed by her condition.

## Conclusions

As explored in this report, the sudden onset of flash pulmonary edema presented in a patient who was treated with electrical cardioversion for an episode of active AF in the setting of heart failure. Despite aggressive efforts, the patient had to be mechanically ventilated for a week until she was able to make a full recovery. This paper intends to shed light on the awareness and importance of unusual clinical complications and the need for a comprehensive and tailored approach for maximized outcomes.

## References

[1] Kannel WB, Wolf PA, Benjamin EJ, Levy D (1998). Prevalence, incidence, prognosis, and predisposing conditions for atrial fibrillation: population-based estimates. Am J Cardiol.

[2] Trappe HJ (2012). Atrial fibrillation: established and innovative methods of evaluation and treatment. Dtsch Arztebl Int.

[3] Cimen T, Algul E, Efe TH, Sunman H, Yeter E (2017). Flash pulmonary edema: a rare cause and possible mechanisms. Turk J Emerg Med.

[4] Klein HH, Trappe HJ (2015). Cardioversion in non-valvular atrial fibrillation. Dtsch Arztebl Int.

[5] Stampfli T, Monnard S, Müller H (2013). Transient symptomatic severe mitral regurgitation after electric cardioversion of atrial fibrillation. Echocardiography.

[6] Batta A, Hatwal J, Sharma YP (2024). Assessment of coronary artery disease in non-valvular atrial fibrillation: is this light at the end of the tunnel?. Vasc Health Risk Manag.

[7] Camm AJ, Kirchhof P, Lip GY (2010). Guidelines for the management of atrial fibrillation: the Task Force for the Management of Atrial Fibrillation of the European Society of Cardiology (ESC). Eur Heart J.

[8] Rimoldi SF, Yuzefpolskaya M, Allemann Y, Messerli F (2009). Flash pulmonary edema. Prog Cardiovasc Dis.

